# Implementation of a Prospective Birth Cohort for Newborn Screening and Early Linkage to Comprehensive Sickle Cell Disease Care in a Low-Resource Setting

**DOI:** 10.3390/ijns12020042

**Published:** 2026-06-16

**Authors:** Umma A. Ibrahim, Aisha B. Musa, Oiza O. Aliu-Isah, Hauwa A. Inuwa, Zubaida L. Farouk, Khadija Bulama, Aisha Mukaddas, Khadija Kamal, Rifkatu N. Auta, Nafiu Hussaini, Aisha A. Galadanci, Yusuf D. Jobbi, Bilya S. Musa, Yvonne Carroll, Lauren J. Klein, Ibrahim M. Idris, Michael R. DeBaun, Muktar H. Aliyu

**Affiliations:** 1 Department of Pediatrics, Bayero University/Aminu Kano Teaching Hospital, Kano 700233, Nigeria; oizaai2@yahoo.com (O.O.A.-I.); faroukzubaida@yahoo.com (Z.L.F.); 2 Department of Pediatrics, Aminu Kano Teaching Hospital, Kano 700233, Nigeria; ayshamus2209@yahoo.com; 3 Department of Medicine, Aminu Kano Teaching Hospital, Kano 700233, Nigeria; sapheena2013@gmail.com; 4 ASPIRE Team, Aminu Kano Teaching Hospital, Kano 700233, Nigeria; khadijabulama@yahoo.com (K.B.); aisha1987.as@gmail.com (A.M.); khadijakamal944@gmail.com (K.K.); rifkatunasiru11@gmail.com (R.N.A.); 5 Department of Mathematical Sciences, Bayero University, Kano 700233, Nigeria; nafiu_hussaini@yahoo.com; 6 Department of Hematology, Bayero University/Aminu Kano Teaching Hospital, Kano 700233, Nigeria; amalgala272@gmail.com (A.A.G.); muazamusa@yahoo.com (I.M.I.); 7 Department of Hematology, Bayero University/Aminu Kano Teaching Hospital/Kano Independence Research Center Trust, Kano 700233, Nigeria; yjobbi@gmail.com; 8 Department of Administration, Aminu Kano Teaching Hospital, Kano 700233, Nigeria; bilyasani@yahoo.com; 9 Department of Hematology, St. Jude Children’s Research Hospital, Memphis, TN 38105, USA; yvonne.carroll@stjude.org; 10 Department of Pediatrics, D. Brent Polk Division of Pediatric Gastroenterology, Hepatology, and Nutrition, Monroe Carell Jr. Children’s Hospital at Vanderbilt, Nashville, TN 37232, USA; lauren.klein@vumc.org; 11 Vanderbilt Institute for Global Health, Vanderbilt University Medical Center, Nashville, TN 37232, USA; m.debaun@vumc.org (M.R.D.); muktar.aliyu@vumc.org (M.H.A.); 12 Department of Pediatrics, Vanderbilt-Meharry Center of Excellence in Sickle Cell Disease, Vanderbilt University Medical Center, Nashville, TN 37232, USA

**Keywords:** sickle cell disease, newborn screening, birth cohort, comprehensive care, survival

## Abstract

In sub-Saharan Africa, where approximately 75% of newborns with sickle cell disease (SCD) are born, under-five mortality remains high, partly due to the absence of newborn screening (NBS) and delayed linkage to comprehensive care. We conducted a prospective, quasi-experimental study involving two sequential newborn screening cohorts at Aminu Kano Teaching Hospital (AKTH), Kano, Nigeria (December 2022–December 2025), to evaluate the feasibility of integrating newborn screening (NBS) with early comprehensive SCD care and to identify barriers to enrollment before 3 months of age. Following an initial implementation period with suboptimal follow-up, a structured family-centered enrollment and communication strategy was introduced to improve early linkage to comprehensive care. During the pre-intervention period, 277 newborns were enrolled (33 with SCD and 244 without SCD [NSCD]), with early enrollment (≤3 months) occurring in 46.5% overall, higher among SCD than NSCD infants (72.8% vs. 43.0%). Delayed enrollment (>6 months) was more frequent among SCD infants. Following the implementation of family-centered communication strategies, 60 additional newborns were enrolled (16 SCD, 44 NSCD), and early enrollment increased to 91.7%. These findings demonstrate that low-cost, family-centered communication and tracking strategies can substantially improve early linkage to comprehensive SCD care following newborn screening in low-resource settings. Early enrollment is a critical step toward reducing morbidity and mortality among children with SCD in low-resource settings.

## 1. Introduction

In low-income settings, where roughly 75% of children with sickle cell disease (SCD) are born, an estimated 50–90% of children with the condition die before five years of age [1]. Newborn screening (NBS) for early identification of children with SCD is the first and most critical step for comprehensive care to prevent morbidity and mortality [2]. The absence of universal NBS in countries like Nigeria is one of the main drivers of high morbidity and mortality among children with SCD [3]. In high-income countries, where NBS is universally implemented and linked to comprehensive care, the survival rate for children born after NBS implementation approaches that of the general population, with estimates as high as 98% [4,5].

In many sub-Saharan African countries, newborn screening for SCD is often the only neonatal screening test performed at birth and is largely limited to urban tertiary centers. Since the 1970s, some pilot NBS programs have been implemented at the subnational level in Nigeria, a country that accounts for nearly half of the approximately 300,000 babies born with SCD each year globally [6]. A recent analysis showed that the annual number of newborns with SCD increased by 13.7% from 2000, reaching approximately 515,000 in 2021 [7]. The World Health Organization’s (WHO) Garki project reported a SCD birth prevalence of 2.1% in Nigeria [8]. The American Society of Hematology (ASH)-sponsored six-nation Consortium on Newborn Screening in Africa (CONSA) aims to screen 16,000 newborns annually in Nigeria alone for SCD [9]. However, previous pilot NBS programs in Nigeria and across sub-Saharan Africa have faced significant limitations, including delays in linking diagnosed infants to comprehensive care, limited long-term follow-up, limited integration into routine health systems, and the absence of a contemporaneous healthy comparison group to evaluate mortality and morbidity outcomes [3,10].

Recent reviews continue to highlight persistent gaps in implementation, sustainability, and longitudinal follow-up after newborn screening across sub-Saharan Africa [10]. Emerging African newborn screening initiatives increasingly emphasize the importance of registry-based longitudinal follow-up and structured enrollment into comprehensive care programs [11,12]. In addition, few studies have documented the immediate post-diagnosis barriers faced by infants younger than 3 months, particularly those that impede timely follow-up for SCD-specific comprehensive care, parental anticipatory guidance, and initiation of preventive therapies.

To our knowledge, existing newborn screening studies in sub-Saharan Africa have primarily focused on screening yield, feasibility, or program implementation, with limited longitudinal follow-up and without direct survival comparisons between children with SCD and those without SCD (NSCD) [10,11,12]. To address this gap, we designed a quasi-experimental, prospective, before-and-after-birth, two-birth-cohorts pilot study at Aminu Kano Teaching Hospital (AKTH), Nigeria, to evaluate the feasibility of implementing NBS with early linkage to comprehensive SCD care and to assess barriers to follow-up in a low-resource setting. We hypothesized that integrating a low-cost, family-centered communication and tracking strategy into NBS would significantly increase enrollment by 3 months of age and ultimately improve the survival of infants with SCD identified at birth.

## 2. Materials and Methods

### 2.1. Study Setting

The study was conducted at Aminu Kano Teaching Hospital (AKTH), a major tertiary referral hospital in Kano State, northwestern Nigeria. AKTH serves as a referral center for Kano and neighboring states, handling over 3000 deliveries annually and providing obstetric, neonatal, pediatric, and hematology services [13]. Newborn screening for sickle cell disease was integrated into routine postnatal care services at the hospital during the study period.

### 2.2. Study Design

We conducted a quasi-experimental, prospective, before-and-after two-sequential newborn screening birth cohorts’ study at Aminu Kano Teaching Hospital (AKTH), Nigeria. The study compared outcomes among newborns enrolled during the pre-intervention period with those enrolled after implementation of an enhanced family-centered intervention. All study procedures adhered to institutional and international ethical standards for human research. Ethical approval for the study was obtained from the Aminu Kano Teaching Hospital (AKTH) Health Research Ethics Committee on 12 October 2022 (NHREC/28/01/2020/AKTH/EC/3397).

### 2.3. Participant Eligibility

The pre-intervention cohort consisted of newborns screened at birth and subsequently offered enrollment in a comprehensive follow-up program without enhanced family-centered tracing strategies. During the pre-intervention period (December 2022–December 2023), a total of 277 infants were enrolled and followed up, including 33 with SCD and 244 with NSCD. All live-born newborns delivered at AKTH during the study period were offered SCD screening. Informed consent was obtained from all families approached, resulting in a 100% screening acceptance rate. Newborns with a normal hemoglobin genotype (HbAA) or sickle cell trait (HbAS) were categorized as the NSCD comparison group, as sickle cell trait is typically clinically asymptomatic in infancy and early childhood. Newborns with confirmed SCD genotypes (HbSS, HbSC, or HbSβ-thalassemia) were classified as SCD participants. Infants with a known maternal history of HIV were excluded to reduce potential confounding because HIV exposure may independently affect infant morbidity, mortality, growth, and healthcare utilization. No infants were excluded based on maternal HIV exposure during the study period.

### 2.4. Informed Consent and Family Counseling

Written informed consent was obtained from parents or legal guardians following a verbal explanation of the study in English or Hausa, using ethics committee-approved translated consent forms. Caregiver comprehension was verified by requesting a restatement of key study information before signing or thumb-printing the consent form. Prior to newborn screening, families received counseling regarding sickle cell disease, the purpose of newborn screening, expected timelines for result disclosure, and the importance of early enrollment into comprehensive care.

### 2.5. Newborn Screening Procedures

Newborn screening for sickle cell disease was performed using dried blood spots (DBS), a minimally invasive and ethically appropriate sampling method. DBS samples were stored in a temperature-controlled, access-restricted facility within the AKTH Hematology Research Laboratory and analyzed using a Bio-Rad VARIANT™ HPLC newborn screening system (Bio-Rad Laboratories, Hercules, CA, USA) with manufacturer-recommended system suitability and internal quality control procedures. To ensure analytical reliability and reproducibility, approximately 15% of samples were retested in line with established laboratory validation standards. Residual DBS samples were retained for up to five years solely for quality assurance purposes before secure disposal, in accordance with ethics committee approval. All study data were de-identified, stored on encrypted servers, and shared only under an institutional data-sharing agreement between AKTH and Vanderbilt University. Newborn screening results were typically available within two weeks after sample collection and were communicated to families by telephone.

### 2.6. Follow-Up and Comprehensive Care

Follow-up visits were scheduled every two months for infants with SCD and every six months for infants with NSCD, reflecting differences in clinical care needs and minimizing participant burden. We recognized that differential visit frequency could introduce surveillance bias, particularly for outcomes dependent on healthcare contact and retention. Accordingly, follow-up outcomes were defined using standardized criteria across groups, and analyses focused on time-to-event and cumulative incidence measures rather than visit-dependent counts.

Children with SCD were initiated on oral penicillin V prophylaxis (62.5 mg twice daily) starting at 2 months of age and received daily proguanil (3 mg/kg) for malaria prophylaxis. Adherence was assessed at each visit through caregiver interviews. Immunization status was tracked with emphasis on the timely administration of pneumococcal and *Haemophilus influenzae* type b (Hib) vaccines. All participants received standard pediatric care at AKTH. Follow-up communication with caregivers was conducted primarily through scheduled clinic visits and telephone contact. Families were routinely called to remind them of upcoming clinic visits to facilitate retention in care. Families who missed appointments were contacted using the verified telephone numbers provided at enrollment. No routine home visits were conducted as part of the study because of logistical and resource constraints. The enhanced post-intervention strategy focused on improving caregiver communication and participant traceability rather than implementing home-based follow-up.

### 2.7. Post-Intervention Family Centered Enrollment Strategy

In January 2024, following identification that fewer than half of infants were enrolled into comprehensive care before reaching 3 months of age, targeted, family-centered enrollment strategies were implemented to reduce the time to the first pediatric SCD clinic visit. The post-intervention cohort included newborns screened at birth following the implementation of enhanced family-centered communication and tracing strategies designed to improve early enrollment in comprehensive care. These strategies included obtaining at least two verified telephone contacts per family, including one from a reliable third party (such as a grandparent or neighbor); providing notification of NBS results within two to four weeks of the sample collection; and documenting detailed home addresses using recognizable local landmarks to facilitate follow-up. In addition, study staff underwent retraining on participant tracing procedures, caregiver communication, and the importance of early linkage to comprehensive care.

During the post-intervention period, a new cohort of 60 children was enrolled using a family-centered approach, comprising 16 infants with SCD and 44 with NSCD.

### 2.8. Statistical Analysis

Categorical variables were summarized as frequencies and percentages and compared between groups using Pearson’s chi-square or Fisher’s exact test. Continuous variables (age at enrollment, gestational age) were analyzed using the Mann–Whitney U test because they were not normally distributed. For survival analysis, mortality incidence rates were calculated as the number of events per 100 person-years of follow-up. Comparisons between groups were conducted using the mid-p exact Poisson method with person-time as an offset, and incidence rate ratios (IRR) were reported with two-sided 95% confidence intervals (CIs). Statistical significance was set at *p* < 0.05. Time-to-event outcomes were analyzed using Kaplan–Meier survival curves, and differences between groups were evaluated using the log-rank test.

Given the potential impact of loss to follow-up (LTFU) on mortality estimates, we conducted sensitivity analyses under four prespecified assumptions:

1. An observed (best-case) scenario in which outcomes were based on observed data only;
2. A worst-case scenario in which all infants with SCD who were LTFU were classified as deaths;
3. An asymmetric scenario favorable to SCD, in which all SCD infants who were LTFU were assumed alive, while all NSCD infants who were LTFU were classified as deaths;
4. A symmetric worst-case scenario in which all LTFU infants in both groups were classified as deaths.

Analyses were performed using Stata version 15 (StataCorp LLC, College Station, TX, USA).

## 3. Results

### 3.1. Pre-Intervention Cohort Results

During the pre-intervention period (December 2022–December 2025), 277 infants were enrolled in the newborn screening cohort, including 33 infants with sickle cell disease (SCD) and 244 infants without SCD (NSCD) (Table 1). All eligible families approached for participation consented to newborn screening and enrollment, resulting in a 100% recruitment rate.

### 3.2. Baseline Characteristics

Baseline demographic and clinical characteristics of infants in the pre-intervention cohort are summarized in Table 2. The overall cohort comprised 162 males (58.5%) and 115 females (41.5%). Baseline characteristics were generally similar between SCD and NSCD groups, although unemployment among heads of household was more frequent in families of infants with SCD than NSCD (36.4% vs. 14.3%; *p* = 0.004).

### 3.3. Enrollment and Follow-Up

**Figure 1:**
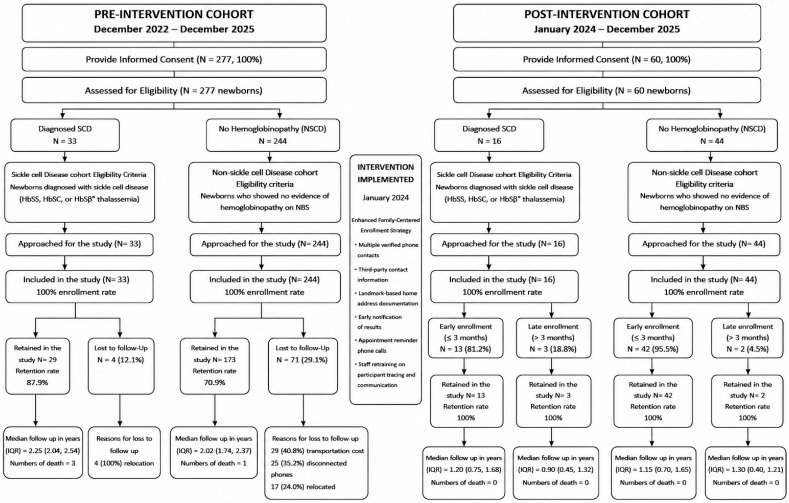
Flow diagram of newborn screening, enrollment into comprehensive care, retention, and follow-up in the pre-intervention and post-intervention cohorts.

Early enrollment by 3 months of age in the pre-intervention cohort was significantly higher among infants with SCD than among those with NSCD (72.8% vs. 43.0%; *p* < 0.001). Overall, 46.5% of infants were enrolled before 3 months of age. Enrollment timing before and after implementation of family-centered communication strategies is summarized in Table 3. Among infants who did not enroll by 3 months, delayed enrollment after 6 months occurred more frequently in the SCD group than in the NSCD group (21.2% vs. 9.0%, respectively). Efforts were made to standardize result disclosure procedures across SCD and NSCD groups, although differential caregiver responses may have influenced enrollment timing. The median follow-up duration was 2.25 years (interquartile range [IQR], 2.04–2.54) for the SCD group and 2.02 years (IQR, 1.74–2.37) for the NSCD group. Retention during follow-up was higher among infants with SCD than among those with NSCD (87.9% vs. 70.9%), while loss to follow-up (LTFU) was significantly lower in the SCD group (12.1% vs. 29.1%; *p* = 0.003) (Figure 1). Attempts were made to contact families of infants classified as lost to follow-up using the telephone numbers provided at enrollment. However, some caregivers could not be reached despite repeated attempts due to disconnected or incorrect telephone numbers. Among NSCD infants lost to follow-up, commonly reported reasons included transportation costs, disconnected telephone lines, and family relocation. In contrast, all infants with SCD who were lost to follow-up had relocated outside the study area.

### 3.4. Pre-Intervention Mortality Patterns

**Figure 2:**
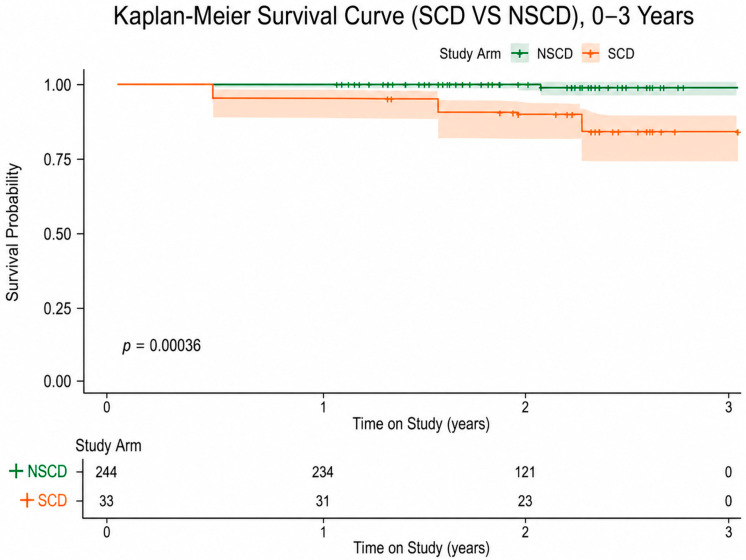
Kaplan–Meier survival curves comparing children with sickle cell disease (SCD) and those without SCD (NSCD).

**Figure 3:**
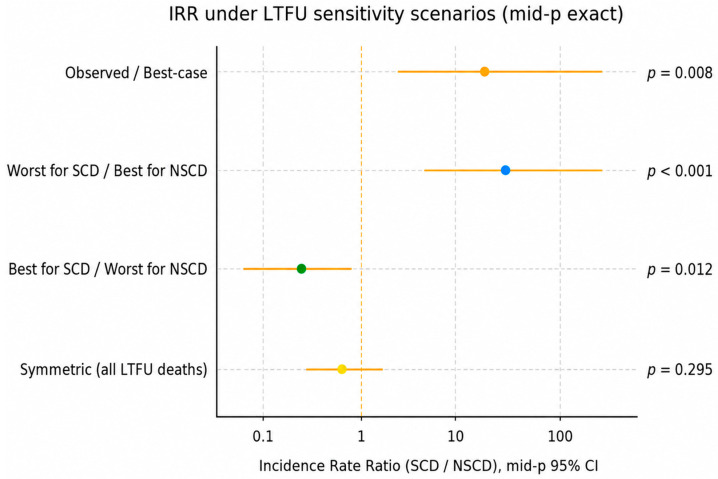
Sensitivity analysis of mortality rates under alternative assumptions for loss to follow-up in the birth cohort. Colored dots indicate the point estimate for each sensitivity scenario.

Over the 2.5-year follow-up period, four deaths were recorded, including three among children with SCD and one among children with NSCD. Most deaths occurred at home and were associated with febrile illness. Among the children with SCD who died, two had caregiver-reported febrile illness prior to death occurring at home, while one child presented with severe anemia before death. The child in the NSCD group who died was also reported to have experienced febrile illness prior to death. Reported ages at death ranged from infancy to early childhood. Caregiver-reported use of insecticide-treated bed nets was documented in most households of children with SCD who died. However, detailed socioeconomic, environmental, and malaria-confirmatory diagnostic data were not consistently available for all deceased participants. The mortality incidence rate ratio for SCD compared with NSCD was 20.4 (95% CI 2.2–536.7; *p* = 0.008) (Table 4). Kaplan–Meier analysis demonstrated stable survival in the NSCD group, whereas survival in the SCD group declined to approximately 91.0% at 2.5 years. (Figure 2). Sensitivity analyses conducted under alternative assumptions regarding loss to follow-up consistently demonstrated higher mortality rates among infants with SCD (Figure 3).

### 3.5. Change in Adherence to Insecticide-Treated Net Use During Follow-Up

Adherence to insecticide-treated bed net use was initially very high (92% of caregivers reported that the infant slept under a net the previous night) and remained high, though slightly reduced after one year (85%) (Supplementary Table S2).

### 3.6. Immunization Coverage Among SCD and NSCD Infants

Routine childhood immunization uptake was high in both SCD and NSCD infants, with most vaccines achieving completion rates of 75–95%. For core antigens, including BCG, OPV, HBV, DPT, rotavirus, pneumococcal conjugate vaccine (PCV), and *Haemophilus influenzae* type b (Hib), no statistically significant differences were observed between groups. Importantly, none of the infants with SCD were completely unvaccinated for any multidose vaccine series. When vaccination was incomplete in the SCD group, it was typically partial rather than absent. Measles vaccination coverage remained relatively low in both cohorts (36.4% in SCD vs. 32.8% in NSCD; *p* = 0.54). The only antigen with a statistically significant difference between groups was the meningococcal A conjugate vaccine (MenA), for which infants with SCD had higher completion rates than their NSCD peers (72.7% vs. 52.0%; *p* = 0.03). This comparison was exploratory because MenA is a single-dose vaccine; higher completion may reflect differential clinic contact rather than sustained adherence to immunization schedules, and should therefore be interpreted with caution (Supplementary Table S3).

### 3.7. Post-Intervention Cohort Results

Following the implementation of family-centered enrollment strategies in January 2024, a second cohort of 60 infants was enrolled, comprising 16 infants with SCD and 44 infants with NSCD (Table 1). All contacted families consented to participation, resulting in a 100% recruitment rate.

### 3.8. Baseline Characteristics

Baseline demographic and clinical characteristics of infants in the post-intervention cohort are summarized in Supplementary Table S1. The overall cohort comprised 31 males (51.7%) and 29 females (48.3%). Baseline characteristics were generally similar between SCD and NSCD groups.

### 3.9. Enrollment and Follow-Up After Intervention

Implementation of the family-centered intervention substantially improved early enrollment into comprehensive care. Enrollment timing before and after implementation of family-centered communication strategies is summarized in Table 3. Overall enrollment before 3 months of age increased from 46.5% in the pre-intervention cohort to 91.7% in the post-intervention cohort (*p* < 0.001). Among infants with SCD, early enrollment increased from 72.8% in the pre-intervention cohort to 81.2% in the post-intervention cohort. A greater improvement was observed among infants with NSCD, where enrollment before 3 months increased from 43.0% to 95.5%. The median follow-up duration was 1.89 years (IQR, 0.93–2.15) among infants with SCD and 2.24 years (IQR, 1.49–2.30) among infants with NSCD in the post-intervention cohort. Retention in the post-intervention cohort was 100% for both infants with SCD and those with NSCD, with no loss to follow-up recorded during the study period (Figure 1).

### 3.10. Post-Intervention Mortality Patterns

Because the post-intervention cohort was enrolled later in the study period, its follow-up duration was shorter than that of the pre-intervention cohort, limiting direct comparisons of mortality outcomes between cohorts. Therefore, no formal mortality comparisons were performed between the two cohorts because of differences in observation periods.

## 4. Discussion

Implementing newborn screening for sickle cell disease, coupled with initiation of comprehensive care before 3 months of age, which is standard practice in high-income countries, has not been widely translated into routine clinical practice in sub-Saharan Africa and has primarily been limited to pilot or externally supported programs, including successful single-site initiatives such as the prospective newborn screening program implemented in Luanda, Angola [1,10,14]. To address this gap in medical care, we conducted a quasi-experimental before-and-after birth-cohort pilot study of infants with SCD and NSCD identified at birth. A contemporaneous comparison group was included to assess morbidity and mortality. Initially, our pilot study revealed poor enrollment within 3 months in both the SCD and NSCD cohorts, limiting the assessment of NBS benefits. By 3 months of age, only 47% of eligible infants were enrolled, a rate too low to demonstrate the potential benefits of NBS. Therefore, we implemented bundled strategies that included prompt reporting of results, collection of phone numbers from a reliable third-party contact, detailed addresses to facilitate traceability, and retraining of the team on the importance of traceability. Collectively, these strategies increased enrollment by 3 months of age, from an initial 47% to about 92%, with complete retention in the post-intervention cohort. Importantly, these interventions were simple, low-cost, and feasible within existing healthcare infrastructure, suggesting that similar family-centered enrollment strategies could be scalable across other low-resource settings in sub-Saharan Africa.

To our knowledge, this is one of the first contemporaneous birth cohorts in sub-Saharan Africa to follow infants with SCD and NSCD from birth, with the aim of improving early enrollment and eventually survival in children with SCD identified by NBS, unlike other birth cohorts in sub-Saharan Africa, such as those in Abuja, Nigeria (2020) and Kilifi, Kenya (2019) [3,15]. Our study design resulted in high early enrollment and retention rates. The Kilifi birth cohort included infants with SCD but lacked a comparison group [15]. The Abuja cohort enrolled infants with SCD from immunization clinics and achieved approximately 75% enrollment in a free prophylaxis program. However, the absence of a comparison group and limited linkage to comprehensive care exposed the challenges of sustaining follow-up in that setting. Most enrolled children were ultimately not linked to a comprehensive care clinic, underscoring this limitation [3].

Recent African newborn screening initiatives increasingly emphasize the importance of registry-based longitudinal follow-up and structured enrollment into comprehensive care programs to improve retention, long-term outcome assessment, and program sustainability [11,12]. Participation in established regional registries, such as the SickleInAfrica Registry, could further strengthen longitudinal data collection, facilitate harmonized follow-up approaches, and improve the comparability of outcomes across African settings. Other NBS programs in Africa have faced challenges, including delayed initial contact with families, limited linkage to comprehensive SCD care, and short follow-up periods, often less than one year [16,17]. These shortcomings may result in an underestimation of true SCD mortality rates within screened cohorts, as many SCD-related deaths occur within the first few months of life when timely interventions are lacking. Collectively, these factors contribute to the likelihood that mortality among infants identified through NBS programs is underestimated, particularly for deaths occurring before four months of age. Vichinsky et al. showed that when penicillin prophylaxis and parental anticipatory guidance are not initiated by 3 months of age, infants with SCD may die from otherwise preventable infections or undetected splenic sequestration [18]. Similarly, a recent observational study in Senegal demonstrated that early neonatal screening significantly improved clinical outcomes [19].

Routine childhood immunization coverage was generally high in both groups, with completion rates exceeding 75% and up to 95% for most vaccines. This level of uptake is comparable to, or exceeds, that reported in other NBS pilot programs in sub-Saharan Africa [15,16]. Although immunization was not a primary outcome of this study, these findings suggest that successful early linkage to comprehensive care following newborn screening may support adherence to preventive healthcare services among infants with SCD. Given the increased vulnerability of children with SCD to severe bacterial infections, integration of vaccination counseling into early post-screening care pathways remains an important component of comprehensive SCD care.

This study was not designed to determine cause-specific mortality. Four deaths were recorded during follow-up, including three among children with SCD and one among children with NSCD. Among children with SCD, two deaths were associated with caregiver-reported febrile illness occurring at home, and one with severe anemia prior to death. The child in the NSCD group who died also reportedly had a febrile illness before death. Deaths occurred between approximately 6 months and 2 years of age. Caregiver-reported use of insecticide-treated bed nets was documented in most affected households; however, detailed malaria diagnostic data, household socioeconomic characteristics, and environmental exposure information were not systematically available for all mortality cases. Most deaths occurred outside formal hospital evaluation; therefore, definitive causes of death could not be established. These observations should therefore be interpreted cautiously and considered hypothesis-generating only. Nevertheless, these findings underscore the importance of comprehensive preventive care models in malaria-endemic settings that integrate malaria prevention, infection prophylaxis, vaccination, prompt fever management, and access to transfusion services [20,21,22,23]. Although preliminary, these observations prompted reinforcement of caregiver education on fever recognition, adherence to antimalarial and penicillin prophylaxis, and timely completion of early childhood vaccinations during routine clinical encounters.

A major strength of this study was the successful implementation of low-cost, family-centered communication and participant-tracking strategies that substantially improved early enrollment and retention following newborn screening. Furthermore, a key strength of this study is the high recruitment rate in a prospective cohort of infants with SCD and NSCD, which supports the study’s expansion. This was a major rationale for the pilot study. Furthermore, we demonstrated to AKTH leadership the importance and feasibility of the NBS program, resulting in its inclusion in the AKTH antenatal care package as standard care to ensure long-term sustainability. Newborn screening for SCD is done on all newborns at AKTH. If a diagnosis of SCD is confirmed, they are enrolled in a comprehensive SCD clinic that offers hydroxyurea and transcranial Doppler screening starting at 2 years of age.

This study has some limitations. The observational before-and-after design precludes causal inference; observed associations should therefore be interpreted as descriptive and hypothesis-generating rather than confirmatory. Residual confounding due to demographic differences between the pre-intervention and post-intervention cohorts cannot be excluded, given the observational study design. Additionally, loss to follow-up was initially substantial in the NSCD cohort during the pre-intervention phase, with a retention rate of approximately 70%, potentially influencing estimates of enrollment and follow-up. Identification of this limitation prompted targeted corrective measures, including intensive staff education and enhanced patient-tracking strategies, which substantially improved early follow-up in the post-intervention cohort. Finally, given that this is a single-center pilot study with a relatively small sample size, our mortality findings should be interpreted with caution. They may not be generalizable to other settings.

In summary, the principal findings of this study relate to the successful implementation of low-cost, family-centered communication and participant-tracking strategies that substantially improved early enrollment and retention following newborn screening. These strategies included the collection of multiple verified telephone contacts, early communication of screening results, detailed address documentation, appointment reminder phone calls, and staff retraining on participant tracing and caregiver communication. Following implementation of these strategies, early enrollment before 3 months of age increased from 46.5% to 91.7%, with complete retention in the post-intervention cohort. These interventions have now been incorporated into the standard of care at our institution, independent of external funding, and may provide a practical implementation model for strengthening early linkage to comprehensive care for infants with SCD in similar low-resource settings. Beyond SCD, these findings highlight how structured communication, participant-tracking systems, and longitudinal follow-up approaches may strengthen newborn screening programs and, more broadly, pediatric preventive care delivery in low-resource settings. Although mortality outcomes were examined, the study was not powered to draw definitive conclusions about mortality differences, and these findings should be interpreted with caution and considered hypothesis-generating only.

## Figures and Tables

**Table 1:** Enrollment of infants in the newborn screening cohort before and after implementation of family-centered communication strategies.

Study Period	Total Enrolled (n)	SCD (n)	NSCD (n)
Pre-intervention	277	33	244
Post-intervention	60	16	44
Total	337	49	288

**Table 2:** Baseline socio-demographic and clinical characteristics of SCD and NSCD infants in the pre-intervention birth cohort.

Variable	Overall (N = 277) ^1^	SCD (N = 33) ^1^	NSCD (N = 244) ^1^	*p*-Value ^2^
Sex of Child				0.153
Female	115 (41.5%)	18 (54.5%)	97 (39.8%)	
Male	162 (58.5%)	15 (45.5%)	147 (60.2%)	
Child Weight at Birth (kg)	3.20 (3.00–3.50)	3.00 (2.90–3.30)	3.20 (3.00–3.50)	0.099
Age at Enrolment (days)	123 (89–156)	65 (44–128)	128 (98–157)	<0.001
Age at Enrolment				<0.001
<6 weeks	7 (2.5%)	6 (18.2%)	1 (0.4%)	
6 weeks–3 months	122 (44.0%)	18 (54.5%)	104 (42.6%)	
4 months–6 months	119 (43.0%)	2 (6.1%)	117 (48.0%)	
Greater than 6 months	29 (10.5%)	7 (21.2%)	22 (9.0%)	
Gestational Age at Birth				0.277
<37 weeks	35 (12.6%)	6 (18.2%)	29 (11.9%)	
≥37 weeks	242 (87.4%)	27 (81.8%)	215 (88.1%)	
Year of Enrolment				<0.001
2022	39 (14.1%)	12 (36.4%)	27 (11.1%)	
2023	238 (85.9%)	21 (63.6%)	217 (88.9%)	
Marital Status of Primary Caregiver				>0.999
Never married	1 (0.4%)	0 (0.0%)	1 (0.4%)	
Married	274 (98.9%)	33 (100.0%)	241 (98.8%)	
Divorced/separated/widowed	2 (0.7%)	0 (0.0%)	2 (0.8%)	
Primary Caregiver				>0.999
Father	2 (0.7%)	0 (0.0%)	2 (0.8%)	
Mother	275 (99.3%)	33 (100.0%)	242 (99.2%)	
Age of Primary Caregiver (years)	29.4 (25.4–34.1)	31.7 (26.9–35.9)	29.1 (25.3–33.7)	0.096
Educational Status of Head of Household				0.207
None/primary/secondary	58 (21.6%)	11 (33.3%)	47 (19.9%)	
Diploma or equivalent	43 (16.0%)	4 (12.1%)	39 (16.5%)	
First degree or higher	168 (62.5%)	18 (54.5%)	150 (63.6%)	
Ethnicity				0.401
Hausa	215 (77.6%)	28 (84.8%)	187 (76.6%)	
Others	62 (22.4%)	5 (15.2%)	57 (23.4%)	
Employment status of Head of Household				0.004
Unemployed	47 (17.0%)	12 (36.4%)	35 (14.3%)	
Employed	230 (83.0%)	21 (63.6%)	209 (85.7%)	

Data are presented as ^1^ n (%) or median (interquartile range). Age at enrollment is reported in days to reflect early infancy, while age categories are presented in months for descriptive and clinical relevance. ^2^ *p*-values were calculated using Pearson’s chi-squared test, Wilcoxon rank-sum test, or Fisher’s exact test, as appropriate.

**Table 3:** Age at enrollment before and after implementation of family centered communication strategies.

Age at Enrollment	Pre-Intervention (N = 277) n (%)	Post-Intervention (N = 60) n (%)	SCD Pre (N = 33) n (%)	NSCD Pre (N = 244) n (%)	SCD Post (N = 16) n (%)	NSCD Post (N = 44) n (%)	*p*-Value *
<6 weeks	7 (2.5)	1 (1.7)	6 (18.2)	1 (0.4)	1 (6.2)	0 (0.0)	
6 weeks–3 months	122 (44.0)	54 (90.0)	18 (54.6)	104 (42.6)	12 (75.0)	42 (95.5)	
4–6 months	119 (43.0)	5 (8.3)	2 (6.1)	117 (48.0)	3 (18.8)	2 (4.5)	
>6 months	29 (10.5)	0 (0.0)	7 (21.2)	22 (9.0)	0 (0.0)	0 (0.0)	
≤3 months (early enrollment)	129 (46.5)	55 (91.7)	24 (72.8)	105 (43.0)	13 (81.2)	42 (95.5)	<0.001
>3 months (late enrollment)	148 (53.5)	5 (8.3)	9 (27.2)	139 (57.0)	3 (18.8)	2 (4.5)	

* *p*-value reflects comparison of early enrollment (≤3 months) before and after intervention using Fisher’s exact test.

**Table 4:** Mortality Incidence and incidence rate ratio (IRR) in SCD and NSCD in the pre-intervention birth cohort using mid-p exact Poisson method.

Group	Events (n)	Person-Years	Incidence Rate per 100 PY	95% CI	IRR	95% CI	*p*-Value
SCD	3	69	4.33	(1.10, 11.79)	20.38	(2.17, 536.69)	0.008
NSCD	1	470	0.21	(0.01, 1.05)	Ref		

Rates are per 100 person-years, with 95% CIs estimated using the mid-p exact Poisson method. IRR and two-sided *p*-value are from a mid-p exact conditional test on total events (median-unbiased). NSCD served as the reference group for IRR estimation.

## Data Availability

The data supporting the findings of this study are available from the corresponding author upon reasonable request and subject to institutional data-sharing agreements between Aminu Kano Teaching Hospital and Vanderbilt University.

## Supplementary Materials

The following supporting information can be downloaded at: https://www.mdpi.com/article/10.3390/ijns12020042/s1. Table S1: Baseline socio-demographic and clinical characteristics of infants in the post-intervention cohort. Table S2: Adherence to insecticide-treated bed net use during follow-up. Table S3: Immunization coverage among infants with sickle cell disease and infants without sickle cell disease.
