# Supplementary material for: Implementation of a Prospective Birth Cohort for Newborn Screening and Early Linkage to Comprehensive Sickle Cell Disease Care in a Low-Resource Setting
Source: Int J Neonatal Screen. 2026 Jun 16;12(2):42. doi: 10.3390/ijns12020042 (PMC13300708; doi:10.3390/ijns12020042)
Supplement: Supplementary file 1 [file IJNS-12-00042-s001.zip › IJNS-4246164-supplementary.pdf]

Supplementary Tables (S1–S3)

Table S1: Baseline socio-demographic and clinical characteristics of SCD and NSCD infants in the post-intervention birth cohort

Variable	Overall (N = 60)[1]	SCD (N = 16)[1]	NSCD (N = 44)[1]	p-value[2]
**Sex of Child**				0.763
Female	29 (48.3%)	9 (56.3%)	20 (45.5%)	
Male	31 (51.7%)	7 (43.7%)	24 (54.5%)	
**Child Weight at Birth (kg)**	3.30 (2.90 - 3.50)	3.00 (2.90 - 3.40)	3.40 (3.00- 3.50)	0.443
**Marital Status of Primary Caregiver**				>0.999
Married	60 (100.0%)	16 (100.0%)	44 (100.0%)	
**Primary Caregiver**				>0.999
Father	1 (1.7%)	0 (0.0%)	1 (2.3%)	
Mother	59 (98.3%)	16 (100.0%)	43 (97.7%)	
**Age of Primary Caregiver (years)**	31.1 (29.0 - 35.4)	33.5 (29.6 - 35.4)	30.4 (29.1 - 35.1)	0.442
**Educational Status of Head of Household**				0.356
None/Primary/Secondary	13 (21.7%)	1 (6.3%)	12 (27.2%)	
Diploma or equivalent	11 (18.3%)	6 (37.5%)	5 (11.4%)	
First Degree or Higher	36 (60.0%)	9 (56.2%)	27 (61.4%)	
**Employment status of Head of Household**				>0.999
Employed	60 (100.0%)	16 (100.0%)	44 (100.0%)	

Data are presented *as [1]n (%) or* median (interquartile range).
[2]P-values were calculated using Pearson's chi-squared test, Wilcoxon rank-sum test, or Fisher's exact test, as appropriate.

Table S2: Adherence to insecticide-treated mosquito nets in children with SCD and NSCD pre-intervention cohort

Variable	Measure	Statistical Analysis Outcome
Adherence to ITNs at baseline (n=277)	Proportion adherent to the night before the clinic visit	255 (92.1%)
Adherence to ITNs at 12 months (n=192)	Proportion adherent to the night before the clinic visit	163 (84.9%)

Table S3: Family adherence to routine childhood immunizations among children with SCD and NSCD under 2 years of age in the pre-intervention cohort

**Vaccine**	**Adherence status**	**NSCD (N = 244)** **n (%)**	**SCD (N = 33)** **n (%)**	**p-value**
**OPV (4 doses)**	None	11 (4.5)	0 (0.0)	0.41**
	Partial	48 (19.7)	8 (24.2)	
	Full	185 (75.8)	25 (75.8)	
**BCG (1 dose)**	None	13 (5.3)	1 (3.0)	0.99***
	Partial	—	—	
	Full	231 (94.7)	32 (97.0)	
**HBV (4 doses)**	None	11 (4.5)	0 (0.0)	0.37**
	Partial	45 (18.5)	8 (24.2)	
	Full	188 (77.0)	25 (75.8)	
**DPT (3 doses)**	None	16 (6.6)	0 (0.0)	0.07**
	Partial	25 (10.2)	7 (21.2)	

	Full	203 (83.2)	26 (78.8)	
**Rotavirus (3 doses)**	None	17 (7.0)	0 (0.0)	0.27**
	Partial	41 (16.8)	7 (21.2)	
	Full	186 (76.2)	26 (78.8)	
**Measles (2 doses)**	None	98 (40.2)	10 (30.3)	0.54**
	Partial	66 (27.0)	11 (33.3)	
	Full	80 (32.8)	12 (36.4)	
**Yellow fever (1 dose)**	None	98 (40.2)	10 (30.3)	0.34***
	Partial	—	—	
	Full	146 (59.8)	23 (69.7)	
**Meningitis (1 dose)**	None	117 (48.0)	9 (27.3)	0.03***
	Partial	—	—	
	Full	127 (52.0)	24 (72.7)	
**PCV (3 doses)**	None	16 (6.6)	0 (0.0)	0.07**
	Partial	25 (10.2)	7 (21.2)	
	Full	203 (83.2)	26 (78.8)	
**Haemophilus influenzae type b (3 doses)**	None	16 (6.6)	0 (0.0)	0.07**

	Partial	25 (10.2)	7 (21.2)	
	Full	203 (83.2)	26 (78.8)	

Values are *n (%)*. P-values were calculated using ** Pearson's chi-squared test or *** Fisher's exact test, as appropriate.
